# The observed relationship between the degree of parasite aggregation and the prevalence of infection within human host populations for soil-transmitted helminth and schistosome infections

**DOI:** 10.1093/trstmh/trac033

**Published:** 2022-04-26

**Authors:** Klodeta Kura, James E Truscott, Benjamin S Collyer, Anna Phillips, Amadou Garba, Roy M Anderson

**Affiliations:** London Centre for Neglected Tropical Disease Research, London W2 1PG, UK; Department of Infectious Disease Epidemiology, School of Public Health, Faculty of Medicine, St Mary's Campus, Imperial College London, London W2 1PG, UK; MRC Centre for Global Infectious Disease Analysis; London Centre for Neglected Tropical Disease Research, London W2 1PG, UK; Department of Infectious Disease Epidemiology, School of Public Health, Faculty of Medicine, St Mary's Campus, Imperial College London, London W2 1PG, UK; MRC Centre for Global Infectious Disease Analysis; London Centre for Neglected Tropical Disease Research, London W2 1PG, UK; Department of Infectious Disease Epidemiology, School of Public Health, Faculty of Medicine, St Mary's Campus, Imperial College London, London W2 1PG, UK; MRC Centre for Global Infectious Disease Analysis; FHI 360, 1825 Connecticut Avenue NW, Washington DC 20009; World Health Organization, Geneva, Switzerland; London Centre for Neglected Tropical Disease Research, London W2 1PG, UK; Department of Infectious Disease Epidemiology, School of Public Health, Faculty of Medicine, St Mary's Campus, Imperial College London, London W2 1PG, UK; MRC Centre for Global Infectious Disease Analysis

**Keywords:** aggregation, schistosomiasis, soil-transmitted helminths

## Abstract

**Background:**

Soil-transmitted helminths (STH) and schistosome parasites are highly aggregated within the human population. The probability distribution of worms per person is described well by the negative binomial probability distribution with aggregation parameter, *k*, which varies inversely with parasite clustering. The relationship between *k* and prevalence in defined populations subject to mass drug administration is not well understood.

**Methods and Results:**

We use statistical methods to estimate *k* using two large independent datasets for STH and schistosome infections from India and Niger, respectively, both of which demonstrate increased aggregation of parasites in a few hosts, as the prevalence of infections declines across the dataset.

**Conclusions:**

A greater attention needs to be given in monitoring and evaluation programmes to find and treat the remaining aggregates of parasites.

## Introduction

The degree of aggregation of dioecious helminth parasites among human host populations plays an important role in the transmission dynamics and sexual reproduction success of parasitic worms.^1^ For soil-transmitted helminths (STH), the parasites reside in the digestive tract and can be expelled through chemotherapy and counted for individuals.^2,3^ In this way, aggregation can be directly assessed, but this is laborious and time-consuming and rarely done outside academic studies. For schistosomes, worms are present in blood vessels and in the lymphatic system and can only be estimated through autopsy. To date, there is only one postmortem study on parasite numbers per person.^4^

Available epidemiological data point to schistosomes, STH (hookworm, *Ascaris* lumbricoides and *Trichuris trichiura*) and filarial worms being distributed across the population according to a negative binomial distribution, which is parameterised by a mean and an aggregation parameter, *k*, which varies inversely with the degree of parasite aggregation. The worm distribution is characterised by the majority of worms being in the minority of the host population. This trend continues under mass drug administration (MDA), with many individuals harbouring no worms after frequent rounds of treatment.

We can also estimate the value of *k* using statistical procedures based on eggs per gram found in faeces (or eggs per 10 ml of urine) and microfilarial counts in blood, which are the surrogate markers of worms per person. Typically, national control programmes conducting STH and schistosomiasis mapping, or impact assessment surveys, collect prevalence and mean intensity data, therefore other methods based on fitting mathematical models of parasite transmission can be employed. These techniques have already been used on STH egg count data. In this paper, we report the results of an analysis of a large egg count dataset from a community cluster randomised trial in Niger studying the impact of various treatment interventions on *Schistosoma haematobium* infection.^5^ Our results show a clear correlation between worm aggregation, as measured inversely by *k*, and infection prevalence in the population. We compare these results with analogous epidemiological data from a STH trial in India and discuss the significance of qualitative and quantitative similarities between the observed patterns.^6^

## Methods

### Epidemiological data

In this study we use two different baseline datasets for schistosome infections (*S. haematobium*) and STH (hookworm) from community cluster randomised trials in Niger and India, respectively.^5,6^ For the quantitative diagnosis of *S. haematobium* infection, the standard urine filtration method was applied.^7^ The technique involves two filtrations of 10 ml of a thoroughly mixed (single midday) urine specimen from each individual, through a Nytrel filter (12–14 mm diameter; mesh size: 20 μm). For hookworm, duplicate slides for each sample were analysed from a single stool sample by Kato-Katz.^7^

### Mathematical model

We employ well established STH and schistosomiasis transmission models, described in detail in previous publications.^1,8^ The equilibrium state of these models is fitted to each cluster in the baseline data, allowing each to have its own aggregation parameter, *k* and basic reproduction number *R_0_*. We obtain the maximum likelihood estimates for the degree of aggregation by fixing values of the density-dependent fecundity (0.005 for *S. haematobium* and 0.01 for hookworm), worm death rate and aggregation parameter for egg output employing estimates from previous studies.^8,9^

## Results and Discussion

**Figure 1.**
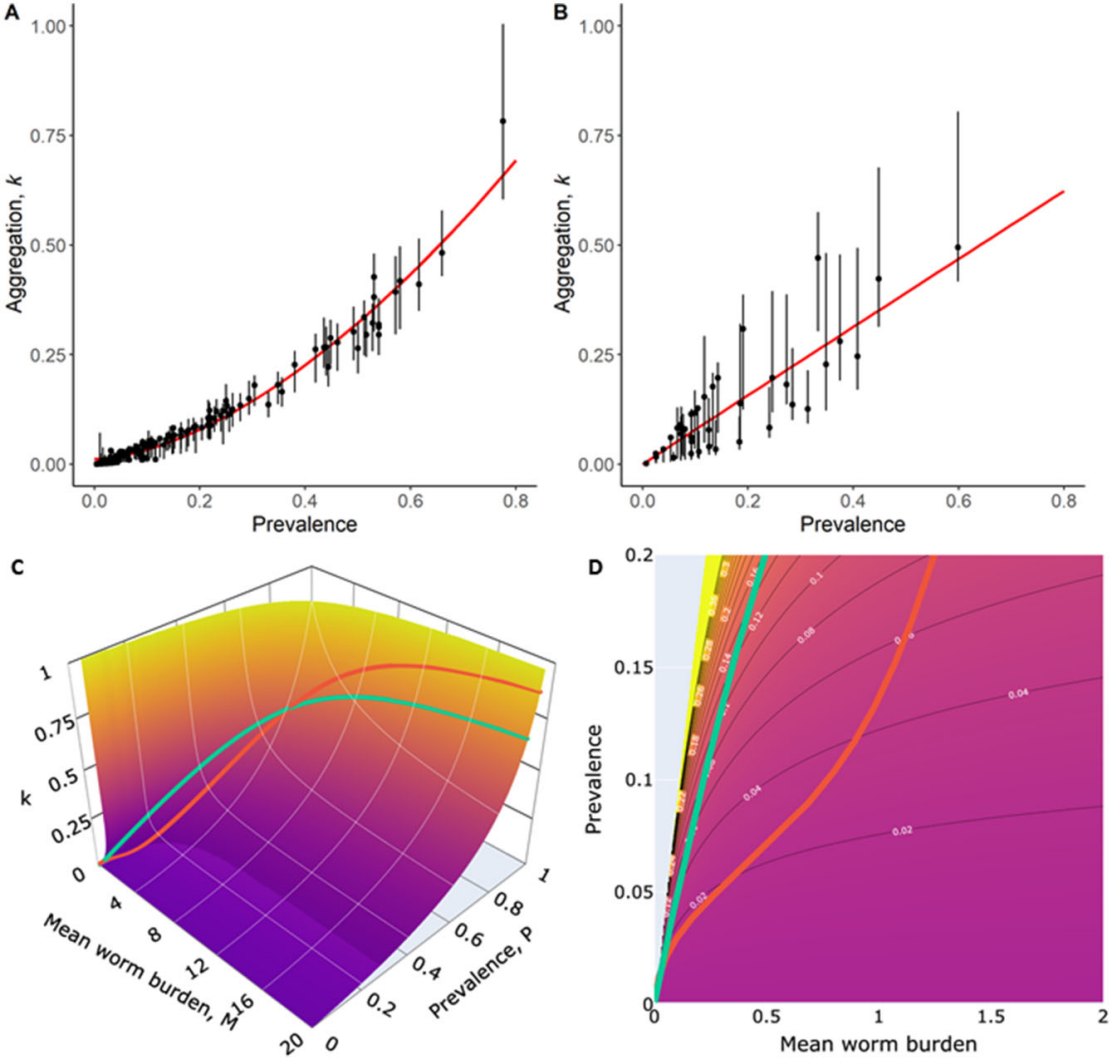
Estimates of the negative binomial aggregation parameter, *k* for individual clusters, derived from baseline data from (A) the Niger study and (B) the India study.^5,6^ Bars represent the 95% credible interval for each inferred value. (C) and (D) display the surface and contours defined by the negative-binomial relationship P = 1 - (1+M/*k*)^–^*^k^* (which broadly represents the relationship between prevalence P, mean worm burden M and aggregation *k*) defined by the negative binomial probability model superimposed with the best-fit lines from (A) red and (B) green.

The results in Figure 1A show a well-defined positive relationship between the measured schistosome infection prevalence in individual clusters from the Niger study and the estimated values of the negative-binomial worm burden aggregation, *k*. This is well mirrored by the pattern observed for hookworm in the India study (Figure 1B). As prevalence declines across the dataset, perhaps due to the impact of repeated MDA, or variation in transmission intensity (as measured by the basic reproductive number R_0_) in different cluster settings, the degree of parasite aggregation rises (*k* gets smaller).^1^

While both parasites are often found in the same parts of the world and often in the same populations, there are significant differences between the life cycles of the two infections. Hookworm is intestinal and is spread directly between human host contact with egg/larval infested soil. Schistosomes are found in the blood vessels and in the lymphatic system where eggs travel to the bladder or intestine and are passed into the urine or stool, which are transmitted via the release of infective stages by the aquatic snail intermediate host in freshwater. The latter implies water contact for transmission to the human host. The close correspondence across both infections in the relationships between the degree of parasite aggregation and the prevalence of infection suggests some commonality in the processes underlying these patterns.

In Figure 1C, we give the best fit lines of *k* vs prevalence, P embedded onto the negative binomial model surface. From empirical observations, it is tempting to ascribe the increased aggregation of parasites under repeated MDA to the existence of a few non-compliers to treatment who sustain transmission in communities via the persistent high worm loads. This is not necessarily the case because Figure 1C shows increased aggregation as prevalence falls, provided the negative binomial probability model well describes observed parasite distributions under changing transmission patterns. As such the observed pattern is more related to the epidemiological mechanism that determines its goodness of fit to the data.^10^ Other factors such as the density dependence of egg production and the mating probability have an indirect effect on worm aggregation by reducing the concentration of infectious material in the reservoir.

To achieve disease control and elimination, the WHO has recommended treatment guidelines based on infection prevalence in a population. Mean worm burden is arguably a proxy for disease burden, but Figures 1C, 1D show that the relationship between prevalence and worm burden is far from linear. In particular, prevalence >50% tells us very little about worm burden. An approximately linear relationship between prevalence and mean worm burden is only found when prevalence is low.

The impact of MDA treatment on aggregation is not clear, specifically whether the patterns of aggregation and prevalence observed are ‘natural’ or the result of prior rounds of treatment. Data analysed here were collected at baseline prior to the implementation of any community-wide treatment interventions. As such, there is some justification in claiming that the relationship between prevalence and aggregation is not influenced by treatment pressure across the community. The implications of the well-defined relationships between prevalence and parasite aggregation as prevalence falls is that much greater attention needs to be given in monitoring and evaluation programmes within low prevalence settings to find and treat the remaining aggregates of parasites.

## Data Availability

All relevant data are within the manuscript.
